# Patient and clinician experiences of remote consultation during the SARS-CoV-2 pandemic: A service evaluation

**DOI:** 10.1177/20552076221115022

**Published:** 2022-08-04

**Authors:** Sue Schutz, Helen Walthall, Joanna Snowball, Raluca Vagner, Nicola Fernandez, Emilia Bartram, Clair Merriman

**Affiliations:** 1 Oxford School of Nursing and Midwifery, 6395 Oxford Brookes University, Oxford, UK; 2 Nursing and Midwifery Research and Innovation, 552380 Oxford University Hospitals NHS Foundation Trust and Oxford Biomedical Research Centre, Oxford, UK; 3 6397 Oxford University Hospitals NHS Foundation Trust and Oxford Biomedical Research Centre, Oxford, UK

**Keywords:** SARS-CoV-2, health services administration and management, organisation of health services, quality in health care, patient, nurse, physician, allied health personnel

## Abstract

**Objectives:**

During the SARS-CoV-2 pandemic, clinicians were instructed to move all but emergency consultations to remote means to reduce the spread of the virus. The aim of this study was to evaluate patients’ and clinicians’ experiences of moving to remote means of consultation with their health care professionals during the SARS-CoV-2 pandemic.

**Methods:**

The study design was a qualitative service evaluation. Twenty-six clinicians and forty-eight patients who met the inclusion criteria consented to be interviewed. Clinician participants were from either medical, nursing, or allied health professional backgrounds. Patients were recruited from diabetes, acute care, and haematology and cancer areas. Data analysis was conducted using a thematic analysis framework.

**Results:**

Following coding and thematic analysis of the data collected from clinicians, five themes were identified: personal and professional well-being; providing a safe and high-quality experience; adapting to a new way of working; making remote consultations fit for purpose and an awareness of altered dynamics during consultation. Patient data was coded into 3 themes: remote consultation adds value; remote consultation brings challenges and concerns about remote consultation.

**Conclusions:**

Clinician and patient experiences reported here are reflected in the literature. The study indicates that remote consultation is not suitable for all patients and in all contexts. Whilst maintaining the benefits to patients, remote means of consultation needs organisational support and preparation. A way forward that maintains the benefits whilst addressing concerns seems urgent.

## Introduction

Remote consultations between clinicians and patients are today technologically possible and are being used in clinical settings alongside or replacing face-to-face.^1^ Remote consultations commonly use two modes of technology, telephone or video platforms which include FaceTime, Skype, Zoom, Microsoft Teams or Attend Anywhere. In the United Kingdom (UK), the National Health Service (NHS) defines remote consultation as: ‘*an appointment that takes place between a patient and a clinician over the telephone or using video, as opposed to face to face’*.^2^ The digital strategy outlined in the Long Term Plan in England^3^ builds on the success of electronic prescribing now used in 93% of England's GP practices, saving the NHS £136 million in the 3 years from 2013 to 2016 and the NHS e-Referral Service, creating expected savings for the NHS in excess of £50 million a year.^4^

The SARS-CoV-2 pandemic led to an immediate implementation of remote consultations for patients. The hasty implementation in clinical practice meant there was limited preparation or education and training of either clinicians or patients although previous evidence identified the need to prepare both clinicians and patients for this type of consultation.^5–9^ Pre-pandemic, the patients’ perspective appears to have been less evident in the literature than that of the health professional.^10^ Of the studies which explored patient perspectives prior to the pandemic, the care setting remained primary care.^11–14^ In the later stages of the SARS-CoV-2 pandemic, the focus broadened with reports of evaluations of remote consultation in secondary care showing encouraging levels of satisfaction from both clinicians and patients.^15,16^

Studies do show high levels of patient satisfaction with remote consultations, especially when this resulted in speedier contact with a health care professional,^10,17–20^ with timely access, rather than the mode of consultation being the most important to patients.^18^ Current evidence in this area indicates that there may be indicators of patients most likely to find remote consultations preferable to face-to-face, including gender (female) and those with higher patient-activation scores.^21^ However, there are concerns over barriers to those with no or little access to digital devices, language barriers, learning difficulties, and digital illiteracy, the elderly or older adults as they may be reluctant to use digital means for healthcare services which challenge any blanket provision.^22^

The balance needed in order to provide for all encompasses pragmatic concerns and therapeutic concerns. Pragmatic concerns include the convenience of remote consultation alongside the challenge of using technology and equipment in a consultation. Therapeutic concerns are around building therapeutic relationships without face-to-face communication.^23^

This paper reports the findings from an evaluation of both patient and clinician perspectives of the experience of remote consultation during the SARS-CoV-2 pandemic in one NHS Foundation Trust in England. The ‘Remote Consultations’ explored in this study were mostly telephone consultations with a small number of video consultations. We aimed to contribute to the emerging knowledge base by evaluating both clinician and patient experiences in one Trust during a specific period of time.

## Methods

Embedded in contemporary health care as providing evidence for the local practice, service evaluation is a design that allows clinicians to evaluate local practice in order to improve services, build knowledge and assist providers in decision-making.^24^ A defining feature of service evaluation is that it seeks solely to make judgements about current services; it serves to identify the standard of service without reference to a benchmark or guideline and thereby does not involve changes to care or intervention.^25^ The study adopted a service evaluation design as designated by the NHS Trust concerned. Approval for the evaluation was given by the NHS Foundation Trust Institutional Board Ethics approval ID 6567. During the time period in which the evaluation took place, all authors were either employees or honorary employees of the Trust

### Patient and public involvement

There was no public involvement in this study due to its nature and the restraints of the SARS-CoV-2 pandemic. The development of the study question and the outcome measures were informed by priorities and experience in the NHS Foundation Trust and across the UK during the pandemic.

### Primary and secondary outcome measures

The primary outcome of the study was to evaluate the experiences of patients and clinicians in engaging in remote consultation using either telephone or video-conferencing. The secondary outcome measure was to support research into the use of remote consultations and contribute to a Trust-wide evaluation of remote clinics at the study site.

### Setting

The evaluation took place in a large NHS Acute Foundation Trust in England. Participants were drawn from secondary and tertiary care services. At the time of the first lock-down, a Standard Operating Procedure was put into place in the NHS Trust and individual services implemented Attend Anywhere* for the purpose of consultations which, in the judgement of individual clinicians, was to be implemented for all outpatient appointments. In practice, this meant that all but difficult diagnostic and complex treatment planning was moved to remote means, but that each specialty was able to make its own decisions. The policy for consultations with new patients was at the discretion of each service. The patient choice was very limited, with most being informed of the change to their appointments by email or telephone, although the policy was for patients to be able to request a face-to-face appointment if they wished. In practice, this choice was limited due to infection control. This guidance was congruent with that provided at the time by the National Institute for Health and Care Excellence.

Interviews were carried out by four female clinical research fellows with a nursing or allied health professional background. All interviewers had completed National Institute for Health Research Good Clinical Practice and informed consent training; they received education in qualitative interviewing and were supervised by experienced qualitative researchers. The interviewers were not employed in the same clinical specialty as the clinicians and patients they interviewed.

### Participants

#### Clinicians

A purposive sample of potential clinician participants was identified via service leads in the clinical areas and approached via email, providing them with an invitation letter and participant information sheet. Participants did not have a current working relationship with the interviewer but were aware that the interviewer was undertaking a period of secondment. They were contacted after a week of the invitation to see if they wished to participate. Clinicians were invited to take part in a semi-structured interview, those interviewed included 10 nurses, 2 physiotherapists, 10 doctors, 1 physician associate and 3 dieticians, making a total of 26 participants. All clinician participants had the experience of telephone consultations and 17 had both telephone and video experience.

Each participant gave informed consent at the start of the interview and these were conducted between March and May 2021. Table 1 shows the inclusion criteria and Table 2 shows the range of clinicians and which type of remote consultation they had the experience of.

**Table 1.** Clinician inclusion and exclusion criteria.

Inclusions	Exclusions
Clinician with relevant registration	
Clinician regularly practices in clinical context evaluated	Locums or agency staff
Clinician has experience in remote consultation	

**Table 2.** Clinicians by profession and experience of using remote consultation.

Clinician	Profession	Type of consultation
Clinician 1	Nurse	Telephone
Clinician 2	Nurse	Telephone
Clinician 3	Physiotherapist	Telephone and video
Clinician 4	Doctor	Telephone and video
Clinician 5	Doctor	Telephone
Clinician 6	Doctor	Telephone
Clinician 7	Physician associate	Telephone
Clinician 8	Doctor	Telephone and video
Clinician 9	Nurse	Telephone
Clinician 10	Doctor	Telephone and video
Clinician 11	Doctor	Telephone
Clinician 12	Doctor	Telephone and video
Clinician 13	Nurse	Telephone and video
Clinician 14	Nurse	Telephone and video
Clinician 15	Dietitian	Telephone
Clinician 16	Nurse	Telephone and video
Clinician 17	Nurse	Telephone and video
Clinician 18	Nurse	Telephone and video
Clinician 19	Physiotherapist	Telephone and video
Clinician 20	Doctor	Telephone and video
Clinician 21	Doctor	Telephone
Clinician 22	Doctor	Telephone and video
Clinician 23	Dietician	Telephone and video
Clinician 24	Nurse	Telephone and video
Clinician 25	Dietician	Telephone and video
Clinician 26	Nurse	Telephone and video

#### Patients

A purposive sample of patient participants was recruited from a data set of patients who had previously given consent to be contacted for research. The potential participants were approached either via email or telephone and invited to take part in the study if they met the inclusion criteria (Table 3). A total of 48 patient participants took part in the study. Table 4 shows the range of patients and which type of remote consultation they had experienced. 23 participants were female and 25 male; 8 had an acute illness and the remainder had long-term conditions, namely haematological disorders, cancer and diabetes. The age range was 27–85 years, and most identified as White British. 46 had experience of telephone consultations and of these 7 also had the experience of video consultation. 2 had video experience only.

**Table 3.** Patient inclusion and exclusion criteria.

Patient inclusion criteria	Patient exclusion criteria
Adult (age 18 and over) English speaking At least one remote consultation (telephone or video) since March 2020 Able to give informed consent	Attendance at any of the following clinics: Paediatric clinics Transition (from paediatric to adult) clinic Patients unable to give informed consent

**Table 4.** Patients by experience of remote consultation.

Participant ID	Age	Gender	Ethnicity	Clinic attended	Consultation type
**P1**	41	M	White British	Diabetes	Telephone and video
**P2**	72	M	White British	Diabetes	Video
**P3**	47	M	White British	Diabetes	Telephone and video
**P4**	27	F	Indian British	Diabetes	Telephone
**P5**	30	F	White British	Diabetes	Telephone
**P6**	53	F	White British	Diabetes	Telephone
**P7**	71	F	White British	Diabetes	Telephone
**P8**	29	M	White British	Diabetes	Video
**P9**	66	M	White British	Diabetes	Telephone and video
**P10**	45	F	White British	Diabetes	Telephone and video
**P11**	61	M	White British	Acute illness	Telephone
**P12**	85	M	White British	Acute illness	Telephone
**P13**	72	M	Unknown	Acute illness	Telephone
**P14**	67	F	White British	Acute illness	Telephone
**P15**	69	M	White British	Acute illness	Telephone
**P16**	43	F	British	Acute illness	Telephone and video
**P17**	59	M	Unknown	Acute illness	Telephone
**P18**	58	F	Black African	Acute illness	Telephone and video
**P19**	73	M	White British	Haematology	Telephone
**P20**	65	F	White British	Haematology	Telephone and video
**P21**	62	F	White British	Haematology	Telephone
**P22**	66	M	White British	Haematology	Telephone
**P23**	33	F	White British	Haematology	Telephone
**P24**	35	F	White British	Haematology	Telephone
**P25**	67	M	White British	Haematology	Telephone
**P26**	72	M	White British	Haematology	Telephone
**P27**	76	M	White British	Haematology	Telephone
**P28**	58	F	White British	Haematology	Telephone
**P29**	68	M	White British	Haematology	Telephone
**P30**	59	M	White British	Haematology	Telephone
**P31**	50	F	White European	Haematology	Telephone
**P32**	49	F	White British	Haematology	Telephone
**P33**	67	M	White British	Haematology	Telephone
**P34**	55	M	White British	Haematology	Telephone
**P35**	71	F	White British	Haematology	Telephone
**P36**	68	F	White British	Haematology	Telephone
**P37**	66	F	White British	Haematology	Telephone
**P39**	69	F	White British	Haematology	Telephone
**P40**	28	F	Black African	Haematology	Telephone and Video
**P41**	70	F	White British	Haematology	Telephone
**P42**	53	M	White British	Oncology	Telephone
**P43**	75	M	White British	Oncology	Telephone
**P44**	73	M	White British	Oncology	Telephone
**P45**	72	M	White British	Oncology	Telephone
**P46**	51	M	White British	Oncology	Telephone
**P47**	64	F	White British	Oncology	Telephone
**P48**	78	F	White British	Oncology	Telephone

Data were collected remotely between March and May 2021 by individual telephone or video interviews. Interviews were conducted by the same four clinicians. Semi-structured interviews were used to capture a rich range of perspectives and explore, in-depth, the patients’ experiences of attending a remote consultation appointment. The researcher and participants had not met prior to the interview and the participants had no prior knowledge of the background of the interviewer.

#### Data collection

Participating clinicians and patients gave informed consent, recorded verbally at the outset of the interview. Interviews were conducted one to one using Microsoft Teams or telephone, audio-recorded and transcribed using a transcription service. Transcripts were not returned to participants as it was not intended, due to the nature and purpose of the study, to aim for data saturation. No participants dropped out of the study. The interview schedules were constructed following a review of the current literature on remote consultation, published both prior to and during the SARS-CoV-2 pandemic. Interviews followed a semi-structured approach to allow participants to express their experiences in a safe and open environment and so provide a meaningful account of their experiences.^26^ The interview schedules were considered for face validity and adapted to ensure the flow and content of the questions^26^ (See supplementary materials).

#### Clinicians

Interviews with the clinicians lasted between 16 and 103 min. Participants gave demographic information including: clinical speciality, clinical role, type of remote consultations used (Table 3). The interview schedule was pilot tested on Nursing, Midwifery and Allied Health Professional colleagues and consequently additional questions were added regarding the environment in which clinicians were conducting remote consultations and the impact of changed ways on working on their well-being.

#### Patients

Participants chose to have either a telephone or video interview at a time convenient for them. Interviews lasted between 15–33 min. At the start of the interview, participants were asked about their demographic profile, diagnosis, and the mode of remote consultation they had experienced (see table 4). Most of the consultations were via telephone (39/48), 7/48 patients had experienced both and 2/48 video only (see table 4). Verbal informed consent was obtained prior to interviewing commencing.

### Data analysis

The work of Braun and Clarke^26^ guided the data analysis; a process that systematically identified, organised, and offered insight into patterns of meaning across the data. Thematic analysis is an accessible way of uncovering patterns and similarities in qualitative data and can also allow unexpected findings to be highlighted via its open yet systematic and rigorous approach congruent with the COREQ guidelines.^27^ All members of the research team were involved in data analysis.

Thematised by hand using an inductive approach, the data were mapped for meaning in relation to the evaluation question. We set assumptions or presuppositions aside during the analysis period applying reflexivity to our own biases,^26^ particularly where the researchers had their own experiences of remote consultation. Data were thematised by interpretation of the coded data into units of meaning which were grouped into themes.

## Clinician results

The data were thematically analysed into five themes (Table 5):

1. Effect on professional and personal wellbeing;
2. Providing a safe and high-quality experience;
3. Adapting to a new way of working;
4. Making remote consultations fit for purpose;
5. Awareness of altered dynamics.

**Table 5.** Clinician thematic analysis: themes, sub themes and codes.

Theme	Sub-themes	Codes
1. Effect on personal and professional well-being	Personal effects Professional effects	‘Thrown in at the deep end’, loneliness, emotional toil, loss of job satisfaction, isolation, personal impact, work/life balance. Risk and concerns for patients, safeguarding, camaraderie, ‘corridor/ad-hoc conversations’, team changes, team support.
2. Providing a safe and high-quality experience	Technical concerns Practice concerns	Equipment, confidentiality, data protection, data uploading and sharing, quality of internet connection, risk of patients and data getting ‘lost in the system’. Lack of equipment/using personal items, distractions/interruptions, training, standardisation, consent, non-verbal communication, safeguarding patients and staff, loss of physical assessment opportunities, when and how to mitigate risk.
3. Adapting to a new way of working	Administrative changes Changes to the process	Burden, two-way emailing, clinic set up, inappropriate use of clinician time, role of admin staff in decision-making. Working day – running to time, flexibility, increased/changed workload, differences between telephone and video, research participation, impact of technical issues, unclear/sporadic communication with patients.
4. Making remote consultations fit for purpose	The consultation Clinician development	Adapting consultation styles, assessing clinical status, impact of patient anxiety on assessments, new patient vs follow-up, types of patient presentation, appropriateness of remote means of consultation, nature and content of consultation, loss of holistic assessment, a chance for home environment assessment. Opportunities for learning, opportunities for service transformation, engagement/confidence/competence in using remote devices, training needs.
5. Awareness of altered dynamics	With clinicians and teams With patients	MDT communication, adapting communication skills, differs according to discipline/profession, desire/motivation to engage, willingness to share new methods. Building a rapport at a distance, consultation focus/dynamic, inclusion of families, patient preference.

## Patient results

Data were thematically analysed into 3 themes (Table 6):

1. Remote consultation adds value
2. Remote consultation brings challenges
3. Concerns about remote consultation

**Table 6.** Patient thematic analysis: themes, sub themes and codes.

Theme	Sub-theme	Codes
Remote consultation adds value	Added practical value	No travel and no parking challenges, a relief to avoid this stress and the cost of travel and parking Very convenient and largely easy Fits better into working and home life, able to avoid taking time off for appointments, can take calls anywhere and anytime Family/significant others do not have to alter their plans Added-value of video-seeing the clinician
	Added value to the consultation	Being treated as an individual is retained Good level of communication is possible by telephone or video Feeling supported and expectations are met Involvement of other clinicians is easier, can see two at the same time Involving family much easier – they don't have to take time off Taking ownership of consultation-better virtually than face to face Choice may be enabled by timing to suit the patient Works well for routine consultations Not knowing if the clinic is running late, being unable to find out how long to wait for the call, not being updated like you would be in the clinic Being interrupted, being unprepared, home life with children at home can interrupt the consultation, sometimes the call comes early Feeling rushed, knowing the clinic is running late and feeling no time for usual small-talk
Remote Consultation brings challenges	The practical difficulties	Poor internet connection interrupting video calls, losing audio or video, fears about missing important information Not able to see non-verbal cues or communication (telephone), trying to work out what the clinician looks like if not known and then feeling the loss of non-verbal communication Knowing the clinician is important Learning new skills – be articulate; don't fear raising difficult or personal topics; learning how to communicate differently
	The relationship with the clinician	Greater need for a clinician to be open and honest, minimise the feeling of being rushed; giving control of consultation to patient Type of dialogue is changed (‘a conversation’ not possible); difficult to process what is said; not feeling comfortable to ask questions, no small talk. Privacy could be compromised, can others listen in? Is my information safe and secure? Need to find somewhere private Where is the clinician? Is that private? If clinic running late where will I be?
Ongoing concerns about remote consultation	Privacy	Loading up results taken at home – is it accurate? Should I be doing something differently? Feeling of taking on responsibility previously the clinician's Physical examination difficult/impossible Loss of visual assessment
	Safety	Remote consultation is not ‘proper care’ Not appropriate for many consultations or for long-term Will this be how it is for good? Is it a cost-saving exercise? Loss of choice – will I be offered the option of face-to-face appointment?
	Fit for purpose	If I am invited to hospital does this mean it is bad news?

## Clinician themes

### Clinician theme 1: effect on personal and professional well-being

The theme ‘effect on personal and professional wellbeing’ illustrates the sometimes profound effects that switching to remote consultation with patients had on clinicians. They felt ‘thrown in at the deep end’, and experienced feelings of isolation and loneliness, being unable to get the professional and personal support that being with their team everyday usually gave. This took an emotional toll on the clinicians who felt a loss of job satisfaction and struggled to establish a work/life balance. Clinicians felt concerned for patient safety and worried about their safeguarding responsibilities.

### Professional well-being

Clinicians were rarely provided with equipment and support to set up and implement remote consultation and had no training or education in using equipment:

*I think there needs to be a recognition that if staff are being required to work in a different way that has to be supported, in terms of staff time. It's all very well saying, we’ve sent you an email, but all this new technology takes time to learn and become expert with. I think there's a failure to recognise that impact on staff work time. (Clinician 4: doctor; telephone and video)*

Nor did they have support around how to change communication styles to undertake a different form of consultation:

*So, if I’ve never met them, and they’re a new patient, I don't think we have the same relationship. … I think it is harder to form a good relationship with (that) patient group just over the phone, without being able to sit and you know, get to know them properly face to face. (Clinician 25: dietician; telephone and video)*

Concerns for patients’ safety and their obligations in safeguarding were common as they could not be certain that the presence of these vulnerabilities had been communicated to them:

*Yeah, I suppose when it was initially – last March, we were told this was the plan, I was very nervous. It felt completely alien and against what we do as nurses, to be telephoning patients with bad news. (Clinician 16: nurse; telephone and video)*

### Personal well-being

Clinicians missed their usual communication with colleagues, the ‘corridor/ad-hoc conversations’ that provided support when difficult decisions had to be made:

*You learn a lot in your job from being around others. In the office, people asking questions and […] and having a discussion. Whereas you don't get that as much at home and it's harder to learn about new things. (Clinician 23: dietician; telephone and video)*

Yet for others, being able to work from home was a real bonus:

*So, it has given me a bit more flexibility, actually, for following up, especially those who are working. So, it's quite good for that point of view. (Clinician 17: nurse; telephone and video)*

The overall message of this theme is that there were many negative effects of working remotely. But this sometimes led to a lack of work-life balance that was felt to have a negative impact personally and professionally. Largely the participants expressed fear and anxiety about the change and the support of their teams and colleagues. This was not simply a problem of isolation but also one that was seen to impact learning from others, team support and how this might impact patient safety. For some, there was a flexibility that they had not been able to enjoy when seeing patients face to face.

### Clinician theme 2: providing a safe and high-quality experience

At the outset of the pandemic, some clinicians lacked even the most basic equipment to support their patients. They were obliged to use their own personal telephones and computers, worrying about the data security and privacy of these means. Some did not have adequate working environments, had poor internet connections and found it difficult to ensure that patients did not get ‘lost in the system’. With whole families at home, finding a private place to talk with patients was difficult.

Physical assessment and examination became impossible for all but the most critical situations and clinicians were unsure how to mitigate any consequent risks to patient safety. Both clinicians and patients could be distracted during remote consultations and lacked basic skills in communicating virtually.

### Technical concerns

Clinicians had serious concerns about the quality of the online consultation, often exemplified by heightened concerns about the technology they had to use:

*I think that in most cases, you were able to convey quite well the nature of the surgery, perhaps even when it's complex, over the phone, but what you don't have – and then particularly if you’re not doing it via video consultation – is the ability to bring up scans to put in front of people or to do drawings for people to try and break it down into more simple, easy to digest information. (Clinician 14: nurse; telephone and video)*

Clinicians experienced worries about risks to confidentiality and data protection, secure uploading by patients of their medical data:

*There is a lot of problems if we request tests outside of the clinic appointment that we’ve noticed since COVID in that test results don't get back to us, and clinicians don't have time to create a spreadsheet to make sure that all of their results are coming back. (Clinician 2: nurse; telephone)*

*I do live with someone else and I have to make sure he's not in. It feels a bit awkward for him more than for me. Because, you know, it's his home as well. I have to make sure he's not around when I’m speaking to patients. [Clinician 25; nurse; telephone and video)*

Many voiced concerns about using their personal devices to conduct consultations:

*Because to start with people were using their mobile phones, their home telephones to make their calls which is not what we should be doing at all. But I think at the beginning we just needed to desperately continue service and put patient's safety first (Clinician 17: nurse; telephone and video)*

### Practical concerns

Distractions and interruptions were experienced, although it is not possible to know whether this might be due to spouse/partner homeworking or children homeschooling or space difficulties:

*I’m clearly sitting in a cupboard and the light goes out every 20 min. So actually finding a quiet space to have a consultation is a huge challenge here, huge challenge. (Clinician 3: physiotherapist; telephone and video)*

The inability to undertake physical examinations meant that clinicians had concerns about the risk to patients and how to mitigate this:

*There's also the safety side of things. Just thinking about physio, certain things I wouldn't ask people to do unless I was around, challenging balance and stuff. Doing that risk assessment. (Clinician 19: physiotherapist; telephone and video)*

Having to understand non-verbal communication remotely was a real challenge as was the difference in communicating sufficiently to gain consent to treatments and ensuring that there was some standardisation in these procedures:

*It's those messages that you don't get when you’re over the phone, and that two-second silence seems to go on for five minutes when you’re on the phone. (Clinician 16: nurse; telephone and video)*

Overall, clinicians voiced concerns about potentially serious data losses, breaches of privacy and confidentiality that could be caused by a lack of proper equipment and organisational adaptation. These concerns also related to the quality of the medium used to communicate with patients and how this caused them to worry about patients being followed up*.* Non-verbal communication was suddenly lacking, and the actual physical safety of patients was a concern.

### Clinician theme 3: adapting to a new way of working

Clinicians found that setting up virtual clinics was a considerable burden, with emails going back and forwards, sometimes crossing and causing confusion. They became much more involved in a clinic set up and felt that this was an inappropriate use of their time yet sometimes found that administrative staff were taking on clinical decision-making, deciding which patients could safely be ‘seen’ virtually and which should attend face-to-face.

Clinicians’ working days were changed beyond recognition as expectations of patients were unknown. They grappled with running to time, building in flexibility with an increased and changed workload. Many had very different experiences when using telephone or video for consultations which resulted in unclear/sporadic communication with patients.

### Administrative changes

Taking on more of the administration of clinics seemed to the participants to become part of remote consultation and was experienced as a burden and a waste of expertise:

*I’ve got a specialist nurse who spends hours organising who is going to see who or talk to who and that's not the best use of a specialist nurse's time at all. (Clinician 17: nurse; telephone and video)*

Participants reported that the organisation did not appreciate the burden of administering clinics remotely:

*I think there's no way around the fact that for this to work really well and be efficient for patients, it probably needs more admin support not less. So … what the Trust needs to understand that the – just placing more and more and more admin burden on to the consultants doing the clinic is just really not a sustainable model. It's really cost inefficient for the Trust (Clinician 20: doctor; telephone and video)*

### Process changes

Clinicians who ran clinics that contained both telephone and video consultations found these to be very different experiences:

*If you’re doing it over the telephone it's really hard to make sure that they’ve grasped it. Whereas at least on email or video you can – and what I often do is I go, are you on email? Can I send you a summary of that so that we can then clarify. (Clinician 24: nurse; telephone and video)*

Although to an extent the working day was more flexible, there were problems in running to time, with continuous technical difficulties:

*It's just the whole thing becomes – if you’ve got 12 people lined up with appointments in clinic you just can't be spending half an hour trying to sort it all out. (Clinician 20: doctor; telephone and video)*

However, the fact that patients waiting for their remote appointment could wait in their own homes was seen as less stressful than in a full waiting room:

*yesterday I was running an hour late, but actually, the patients are at home. I felt so much better, thinking, okay, they’re at home. They’re in there. They can keep themselves busy. (Clinician 11: doctor; telephone)*

The process of switching to remote consultation brought several concerns to the attention of clinicians in adapting to new ways of working. Many felt they had to do their own administration while lacking both time and skills to do this and the time spent organising consultations was a waste of their expertise. Participants were struggling to cope with outdated equipment – usually their own personal devices and felt that the organisation should provide more administrative and technological support.

### Clinician theme 4: making remote consultations fit for purpose

Clinicians struggled at times to adapt their consultation style to remote means of communication. They were challenged by having to assess the clinical status of patients without seeing them or seeing them only on a screen and were concerned about the impact of patient anxiety on assessments. There were particular concerns about new patients and about what had to be communicated or discussed.

Clinicians did appreciate that there were opportunities for learning, for service transformation, and for them to engage with growing confidence and competence in using remote devices.

### The consultation

This theme illustrated significant concerns about the appropriateness of remote consultation when assessing the clinical status and the impact of patient anxiety on assessments:

*It will be really challenging to pick up the physical signs over the telephone, so I think in that situation, if physical examination is essential and it makes a difference to your clinical judgment, then I find it really not the best type of consultation. [Clinician 22: doctor; telephone and video)*

Who should make the decision regarding which patients needed to be seen face to face and who could be seen virtually was a contentious issue. Some clinicians felt this was a clinical decision, not one for administrators to make and often the debate was around using remote methods for new patients versus follow-up:

*Our secretary will call people or email them to ask them which one they would prefer. I don't know how much information they get about the two different options. I think they might just be asked would you like a telephone appointment or a video appointment. (Clinician 25: dietician; telephone and video)*

The loss of opportunities for full assessment enabled by a face-to-face meeting raised serious safety concerns:

*I guess I used to diagnose a lot of skin cancer because people with chronic leukaemia have an increased risk of skin cancer. So they’d be coming into me expecting a conversation about their blood results and I would be looking at them and saying, gosh, you’ve a funny looking sore on your ear, how long has it been there? … that sort of opportunistic diagnosing other things has gone. (Clinician 21: doctor; telephone)*

Clinicians did discuss the opportunity to see the home and get a sense of the environment in which, the patient lived, which they had not previously been able to do. This somewhat ameliorated the concerns about the feared loss of holistic practice and added a new dimension to the consultation that would have been missed with a clinic face-to-face appointment:

*I think for me what really was poignant was that actually they could have their family. They were in their surroundings; they were sat around their kitchen table or dining room or somewhere in their house that they wanted to be. (Clinician 13: nurse; telephone and video)*

### Clinician development

Several positive experiences were voiced in terms of the learning opportunities remote consultation offered for the clinician such as learning about adapting their consultation styles, reflecting on the appropriateness of remote means of consultation, and the nature and content of the consultation:

*He said, oh, I’ve never been able to talk about it. It's been bothering me for a couple of years, but I’ve never been able to talk about it [personal symptom]. Thank you, because I was able to talk to you. I reflected on this afterwards, and I thought, was it me? Was it anything different that I did compared with anyone else? I thought, no, I think the difference was it was the telephone. (Clinician 13: nurse; telephone and video)*

Clinicians saw an opportunity for service transformation, and for their own training and development in engagement, confidence, and competence in using remote devices:

*So I’ve done, with one of my SpR colleagues – so I was at home and he was seeing a patient here and I dialled in. So we’ve been able to network and do a joint consultation together which worked actually really, really well. We’ve not quite perfected that as a process but we’re working on it. I think that's probably a Trust wide opportunity because of the different specialities. (Clinician 24: nurse; telephone and video)*

This theme illuminated clinicians’ concerns about making remote consultations fit for purpose. There was universal agreement that some face-to-face consultations were needed but that sometimes they were expected to make physical assessments remotely. This raised serious safety issues at times and there was concern about who was making the decision to change a face-to-face consultation to a remote one. However, advantages were experienced, including using remote consultations to see family and home environments.

### Clinician theme 5: awareness of altered dynamics

The subtle and sometimes not-so-subtle alteration in the dynamics of remote consultation was not lost on the clinician participants. They recognised the need to adapt their communication skills and that the success of this endeavour relied upon a willingness to engage with this new world and to share new methods and skills with others. New means of team communication had to be adapted to this seemed to differ slightly depending on the discipline and profession.

New skills included building a rapport with patients at a distance and paying more attention to the consultation focus whilst including families and considering patient preferences**.**

### With clinicians and teams

This sub-theme concerning changes in communication with both the professional team and the patient. Clinicians appreciated their colleagues’ desire and motivation to share new methods to help them to learn fresh skills and conduct remote consultations effectively:

*So it's been a big learning curve, how to handle our clinics and our patients by telephone, I feel. (Clinician 2: nurse; telephone)*

Communicating with the wider multidisciplinary team became challenging and was sometimes impossible, impacting the clinician's own development:

*I very rarely get to attend the MDTs these days. But actually the MDTs were a really good learning … So I always found it quite a good learning environment. I miss that, we don't get that now. (Clinician 2: nurse; telephone)*

The awareness of participating clinicians of an altered dynamic in remote consultations was challenging but also held potential and real benefits. The ability to carry out a meaningful consultation remotely was very much felt to be dependent on establishing trust and rapport with patients. Clinicians found skills that they were previously unaware of and experienced an opportunity to involve families and to enhance patient education and self-management.

### With patients

A significant amount of the data collected from clinicians concerned their worries about how to build and maintain a rapport with patients at a distance:

*So I think when you first meet someone – what I’m very focussed on is building trust and building a relationship, knowing that this patient needs to trust you for the next number of years that they’re going to be seeing you. I think that's more difficult to do on a telephone context. (Clinician 17: nurse; telephone and video)*

At times having a family member on the call was helpful in getting a different perspective and at other times, clinicians were unsure who was listening to the consultation or if the patient was giving the consultation their full attention:

*I can hear people doing things like emptying – you know, the sounds of emptying a dishwasher, or I can hear them tapping on a computer. Yeah, you can hear that they’re doing something else. (Clinician 25: dietician; telephone and video)*

The focus of the consultation was seen as a big factor in success, remote methods of the consultation were not seen as ideal for communication of bad news or diagnoses. Whilst patient preference was seen as a major factor in decisions about the use of remote consultation, clinicians were very aware that there was often not a choice:

*It's really not a good system for when you need really in depth conversations with someone, when you’re talking about a new diagnosis or breaking bad news about outcome of a treatment and that type of thing. (Clinician 4: doctor; telephone and video)*

Clinicians participating came from a wide variety of professional and clinical backgrounds and recognised that these differences will impact the altered dynamic of the consultation and the clinician's consequent experience. Consultations were inherently different and adapting one's approach was part of a successful consultation:

*I feel that, on the phone, I’m an even better active listener. It's interesting, because I can just sit there, and I can just listen, and it is – you’re not worried about your body language and you’re not looking around. You’re not – you’re just totally in, with that patient. (Clinician 11: doctor; telephone)*

## Patient themes

### Patient theme 1: remote consultation adds value

Having appointments moved to remote means was seen by patient participants to often add value to the consultation. This could be seen as both practical advantages and positive experiences.

### Added practical value

By far the greatest change in the experience of care that patients expressed was that there were no travel and no parking challenges involved in seeing their clinician, these being seen as major causes of stress previously. In addition, patient-participants felt that remote means fitted better into their working and home lives, with the whole experience being quicker and easier. They particularly appreciated the ability to include their family or significant others in the call.

Participants identified areas in which remote consultations had positive impacts on their experiences. Being offered a virtual appointment was perceived as a benefit as it meant they did not have to make the journey into a busy town and cope with long commutes (for some,) traffic and the need to queue for parking:

*it's more practical because the hospital is in a very busy part of town, and driving can be a pain, getting a parking space can be a pain, paying for a parking space. Either that or I get a train a train and several buses. So yeah I think for me it makes sense. (Patient 8; telephone and video)*

Participants also reflected on how much easier it was to fit clinics into their home and working lives, enabling them to attend appointments without disruption to their otherwise busy lives:

*Most of your day or half an hour on the phone, then you’ve got the rest of your day. Whereas you go for an appointment and your whole day is taken up with travelling and being at the clinic. (Patient 42; telephone)*

With the option of virtual clinics, patients and their family members could be more easily present, meaning the patient had the added benefit of being in comfortable surroundings with their support structures. Participants generally felt most clinicians could and did involve families in remote consultations:

*I do because especially – I mean I tend to say oh I’m fine, I’m fine, and [husband] will chip in and say well actually you know she has this or this is or whatever. He obviously sees me differently to how I feel sometimes, so it is helpful, yes. (Patient 21; telephone)*

The perceptions of patient participants regarding the use of the telephone rather than a video call showed that some patients were positive and found that the dynamic was similar to video, that they felt supported and that their expectations were being met:

*Overall I was quite happy. As long as we can hear and communicate clearly over a phone, that's fine for me. (Patient 6; telephone)*

Seven participants experienced both telephone and video consultations. These participants reported they mostly favoured video over telephone appointments as they were able to see the clinician:

*I didn't think there were any drawbacks to it. I was I guess lucky, internet connection was good so there was no lag or stutters or anything breaking up the connection, so that was all good. So that was very positive. [Patient 8; video]*

Many patient-participants found the move to remote consultation very convenient and largely easy to do. They found that balancing appointments with their work or day-to-day lives was much easier and that they, their family or significant others did not have to alter their plans to accompany them to the hospital. In particular, remote consultations using video were felt to add value by being able to see their clinician.

### Added value to the consultation

Patient participants largely felt that they were still treated as an individual when the consultation was changed to remote means. They felt that a good level of communication is possible by telephone or video and that thy felt supported and that their expectations were met. Participants valued having met their team face to face previously and being able to put a face to a name. Patient participants reported that somehow the dynamic of consultation was changed but that therapeutic relationships could be maintained:

*I think a big part of good medical care is building good therapeutic relationships … So, if you don't have that opportunity, and you’re not able to do that, for whatever reason, I guess the overall opinion of the experience wouldn't be so positive. I would have felt that I wouldn't have got as good a service as if I had attended face-to-face. (Patient 15; telephone)*

Being able to see or at least visualise the clinician from memory, was important to successfully using remote means of consultation because the interaction depended upon a trusting relationship:

*Because I’ve learnt to trust them, that's a huge part of making even an audio conversation valuable I thought, well, I’ve learnt to trust these people, and because of that high level of trust, an audio works, if you’re with me. If that hadn't been built up, I don't know really. (Patient 25; telephone)*

Although by demonstrating that they were committed to the relationship, clinicians could establish trust without ever meeting the patient:

*During the pandemic situation, again they’ve been there for me and I have – there's a senior nurse who is like my guardian angel and she's in contact by email. I’ve sadly never met her, yet. She's been there looking after me for about – I think it's about two and a half years now. (Patient 13; telephone)*

Compared to experiences of waiting in clinics, the way in which remote consultations were organised during the pandemic meant that there were changed perceptions in the level of control that the patient had:

*No more hanging around with a cup of coffee, waiting and looking at my watch. (Patient 25; telephone)*

Being called at the correct scheduled time was important to the participants and impacted their experience:

*I have to say on both calls it was very prompt. I don't know what time the appointment was. I think it was three o’clock. It may have been one or two minutes after three, but I wasn't waiting hours. (Patient 45; telephone)*

### Patient theme 2: remote consultation brings challenges

It was clear from the patient data that many found challenges in moving to remote consultation. Undoubtedly, much of this was caused by the inability to plan and implement preparation for this move. Patients had both practical challenges to resolve and changes in their relationship with the clinician which had now to be mediated without face-to-face contact.

### The practical difficulties

Several participants reported not receiving their call at the agreed time with a small number reporting it did not happen for a few hours after the scheduled time. Participants were generally understanding of being called late, just like a clinic running late in the hospital. However, the difference with the face-to-face clinic appointment they knew that they were on the list to be seen as they would report to the receptionist. The receptionist would also inform them if the clinical was running late. Consequently, when participants were not called at the allocated time, they were concerned that the consultation would not take place:

*but when they say it's going to be at 4 o’clock, and it then doesn't happen till half-past 6, it can leave you feeling a little bit anxious. (Patient 46; telephone)*

Participants felt it was important they were notified if the appointment was going to be delayed, however, only one participant reported this happening. There were suggestions it might be more appropriate to be given a time window for their appointment, suggesting this would help with times when the clinician was not running to time:

*it would need to be a fixed time, so I can plan my day appropriately around that and make sure that I’m actually free at that time. So, yeah, for me, no. Time frames of – a 4-hour time frame or a 6-hour time frame, no. (Patient 15; telephone)*

A small number reported appointments being several hours early, meaning they were interrupted, could not speak comfortably, or were unprepared for the appointment. This led to a feeling they were being ‘fitted in’ by the clinician, which led to them experiencing feelings of being rushed. They reported this was different from their experiences of face-to-face appointments:

*They have a tendency to – when they’ve got a spare moment, which may be at a completely different time of that day, to call you up just on the off chance of whether you can do it. I think that is inconvenient for everybody because it rather implies that nobody has any other life, and that is not a good situation. (Patient 30; telephone)*

Participants raised a number of concerns about processing information, communicating under pressure and felt clinicians spoke too quickly. Participants reported finding it hard to ask clinicians to slow down or repeat points during remote consultations:

*She could have talked more slowly. Face to face she does talk more slowly. It takes me a while to hear the things, but I do not always process things. If it's something not expecting, I do not always process it quickly. (Patient 48; telephone)*

Participants reported finding it hard to convey symptoms over the telephone or describe a wound or skin condition. This raised concerns they were going to be diagnosed or have treatment based only on their own explanation. They also felt that the lack of visual cues could contribute to misdiagnoses:

*I think when you are talking about things that you cannot physically take a photograph of, things which you cannot express other than to the limits of your ability to articulate it, then you have a problem. (Patient 11; telephone)*

With this in mind, a small number of participants who were being reviewed regularly for long-term conditions reported that remote consultations brought additional burdens to them as they were often asked to provide data prior to an appointment. Such data included blood pressure recordings, blood glucose readings and/or tests to be completed elsewhere or by themselves that would have normally been conducted as part of their consultation. This caused anxiety that the right information was not gathered and a concern this would impact their consultation:

*Oh what's your blood pressure? I said well I don't know. So I went out and bought myself a blood pressure machine. It seems to be putting more and more on to the patient [….] it does not bother me but it's all very well me taking it because I only bought the little cheap machine. Is it as good as the one that the doctors and the hospital use? I don't know. (Patient 7; telephone)*

However, this view changed when there were technical issues with the internet leading to frustration and missed information:

*Technology-wise, the phone conversation tended to be more reliable at the time. I wasn't necessarily going to be at home so I didn't necessarily know if I was going to have great internet for a video call. [Patient 5; telephone]*

All participants agreed that technologies can support remote consultations and worked well for routine test results, straightforward reviews, and regular follow-ups. However, participants felt receiving bad news, discussing sensitive issues, complex treatment plans or needing a physical examination remote consultation were not suitable:

*I think it is probably easier [….] with a long-term condition that just needs monitoring, it's probably very easy. I’m not sure how comfortable I would feel if I had a new problem, where it would require a physical examination. (Patient 1; telephone and video)*

Patient participants reported feeling a lack of choice in what mode of consultation they were offered. Participants felt it would be good to be offered a choice of appointment type, either telephone, video, or face-to-face rather than having to fit in with what the service provided.

*It would be nice to be able to make a selection. because then I would feel a bit more like I was in control instead of it all being done for me (Patient 48; telephone)*

The opportunity to involve family and significant others was welcomed, particularly as this would not be possible during the pandemic. However, participants reported that they felt that clinicians ought to purposely inform them that family members were welcome and support them to take part in remote consultations:

*Yes, I think if all of a sudden I was being given bad news I probably would want my husband in the room with me, so at least if I need to hand over a telephone, I could. Or even if it was a video thing, I could do exactly the same, I can call him, he’d be there. (Patient 43; telephone)*

Like the clinicians, patient-participants perceived remote consultation as impacting the relationship they had with the clinician. Raising difficult or personal topics was more challenging and this had an emotional impact. Patients wanted reassurance and were concerned about privacy, not always wanting their family involved in the exchange. Patients felt that remote means were not the best way to convey bad news or conduct physical examinations, and that this type of consultation was not really ‘proper care’.

### The relationship with the clinician

Essentially the means and nature of the communication with their clinicians had changed for patients during the pandemic. They felt that they had to learn how to communicate differently. The vast majority of consultations moved to telephone rather than video and this brought some challenges for those who wanted to talk about something sensitive, finding that raising difficult or personal topics was dependent on the ability to visualise the clinician:

*If I’m talking about something that's really personal and really intimate and sensitive, eventually I’d want to put a face to a name. (Patient 18; telephone and video)*

And therefore, video provided a better solution over the telephone:

*I think video would definitely be more beneficial for that. Rather than just on the telephone because I think […] it's harder to pick up social cues of who's wanting to talk next. It's still harder than with […] a video than physically in person. I think that's definitely still better than just over the phone. (Patient 5; telephone)*

Participants felt telephone consultations meant that they were unable to interpret body language and facial impressions from the clinician. Patient-participants commented they found it hard to interpret the tone of the conversation over the phone.

*In a face-to-face environment the clinician notices you are uncomfortable or there a bit of hesitation whereas it does not always – it is not easy to convey this over the phone. (Patient 42; telephone)*

An inability to read visual cues meant some participants felt unable to ask questions at the end of the consultations and therefore felt rushed and not listened to. Again, patient-participants reported this was different from their experiences of face-to-face consultations:

*The brain works in terms of you think, oh yeah, I do have some questions, it has not been answered, and you look down on your piece of paper, but by the time you say it, the conversations finished. But if you are in face-to-face situation, you’ve always got those few seconds, maybe a couple of minutes where you’re going through the pleasantries and then you think oh, just one more question. (Patient 15; telephone)*

There was a greater need for clarity and honesty and trust was a key factor in a successful consultation.

*I think that's one of my biggest issues about the type of consultation people have is the imbalance of power, is that at the moment it's not patient choice. (Patient 1; telephone and video)*

Of the patient participants, the vast majority (39/48) had experienced telephone consultation and some participants commented that not knowing what the clinician looked like was a factor that altered the relationship:

*I think having a consultation with a consultant which you have never met and you have no picture of them in your head-there's something very psychological comforting knowing what the person looks like … I would really like to know what he looks like without having to Google him. (Patient 43; telephone)*

Many of the changes experienced in moving to the telephone as a means of consultation were unforeseen, such as the loss of a conversational element:

*But with a face-to-face, it's quite different, because if you’re having a conversation about something, […] the doctors can't really say, now it's time to leave in the middle of a consult. They can run over time a little bit if they have to. But with a telephone you’re thinking, oh, 15 min, prioritise the conversation. (Patient 4; telephone)*

Visual cues and body language were also lost during telephone appointments and patients felt that this impacted negatively on the consultation with the potential to miss vital information. This lack of personal presence was also felt more strongly when discussing subjects of a sensitive nature and feeling uncomfortable about asking questions:

*Talking to somebody over the telephone wasn't the same as when you speak to somebody face to face, only because when you have somebody face to face you can see their emotions. You stop once you can see that person is becoming uncomfortable with the subject. But on the telephone, it's very difficult for that person to see. […]it wasn't comfortable doing that over the phone, to be honest. (Patient 4; telephone)*

And sometimes made it difficult to process what was being said:

*If it's your first telephone appointment, have somebody with you, if possible, so you’ve got a second set of ears, to hear whatever it is. (Patient 47; telephone)*

This meant that participants felt a greater need for a clinician to treat the patient as an individual and be clear, open and honest:

*What might be clear to one person isn't clear to another. It's not quite as easy over the telephone to ask for clarification or to say, I don't understand that. Often, it's the body language that tells us they didn't understand it. (P44; telephone)*

*It's not anything they’ll say on the phone or on the video call that will be any different to what they’re going to be telling you face-to-face, but it's that human touch which shows that they actually know who you are, and that they’re aware of you, and you’re not just an NHS number. (Patient 20; telephone and video)*

Patients felt that demonstrations and explanations could be maintained via video call where it was not possible by telephone:

*It was quite useful for the doctor to show me things on that video call, with regards to where to put the injection, all that kind of stuff, which couldn't be done over the phone. They could tell me, but they couldn't show me. It was far more informative. (Patient 10; telephone and video)*

The emotional impact of changing to remote means of consultation included the inability to find reassurance from talking with patients in a similar situation:

*It doesn't worry me too much at all, you see other people in your situation as well and you can be chatting to them about your own treatment or ….especially at the beginning when you were chatting to other ladies or gentlemen who were going through similar things really. (Patient 46; telephone)*

Patient-participants realised quickly that they had a feeling of responsibility to be able to articulate their symptoms verbally:

*I found it challenging sometimes, because I wanted to get a point over, and always having seen medical professionals face to face, in my past – [I’m 61], that's been quite some time, I then had to learn to articulate what was the condition, what the problem was, and then a lot more information. So I had to use my quick-study methods and then try and articulate correctly any conditions that were a concern to me. (Patient 11; telephone)*

Support, preparation and planning were required to cope with the change in the dynamic of remote consultation. Patients needed to feel that they were able to communicate their health concerns adequately, be treated as an individual and to have the same level of support and reassurance that they would gain from a face-to-face consultation.

### Ongoing concerns about remote consultation: privacy, safety and fit for purpose

Patient-participants articulated a number of worries about changing to remote consultation. There was a feeling that they had no choice over how they communicated with their clinician. Whilst understanding the reason for the change, some patients felt that they had not been involved in the decision about their particular arrangements. Downsides of remote consultation were voiced too including that not knowing that the clinic is running late, being interrupted or unprepared, not able to see non-verbal cues and at times feeling rushed.

Given that the choice was not really theirs, patients were able to identify the best means for certain types of consultations and felt that certain people might be more reassured by seeing a clinician face to face:

*Obviously it wouldn't suit everybody, you might get certain people who don't like to talk on the phone, or the video, or maybe that they just like to talk in person, like to see somebody. Perhaps they feel more reassured than talking on the phone, I don't know. (Patient 48; telephone)*

Being reassured could be achieved virtually if the opportunity to ask follow-up questions was available. Patient participants could clearly express the nature of consultations suitable for remote means:

*I think when it hasn't worked has been when we’ve tried to talk about new treatment and ideas where it's got a bit confusing and then the inability to catch someone's eye or just the body language, it does get in the way just as it would with any other phone call. However, I think as I said in various other contexts, it's a very sensible thing. I’m trying to think about what the split of those for me were. I think probably about 60/40. I think about 60% of my conversations, meetings, appointments are catch-up-type ones which would potentially move to the phone without any major problem. (Patient 41; telephone)*

In particular, patients were very clear that remote means are not the best context for giving bad news:

*If you were upset – I mean, it must be awful to – if you gave bad news over a video call and you could see the person on their own being upset and it's just – if you were actually there in person, it would just be a hand on the arm or something like that but if it's a video call, you couldn't do it. It would be very difficult to reassure somebody*. *(Patient 43; telephone)*

Patient-participants imagined that being called in for a face-to-face meeting may be bad news:

*But then the problem is, and this has actually happened, one of the support groups that I’m a member of, somebody said, I’m really worried, they’ve said I’ve got to go in for a face-to-face consultation. Does that mean I’ve got bad news? (Patient 19; telephone)*

Consistent with this was the notion that any discussions that involved decisions about the future treatment would be the best face to face:

*I think if there was something serious, say if my PSa levels really rose and I think I would have to have an appointment to go back up and then I think personally it would be to see what treatment I would need and how things were in the future. I think we would – and my wife would – it's just nice sometimes then to sit down. (Patient 48; telephone)*

There were fears that the remote means of consultation for them had not been successful and that it would have been much more likely that their health problem would be resolved should they have seen a clinician face to face. Patient-participants voiced fears about missed diagnoses that could be picked up with a face-to-face appointment but not remotely:

*I wouldn't have known that was a problem if it hadn't have been from the accidental picking up by the test in haematology, just by listening to my heart. So obviously, there is less chance of picking up those accidental finds if it's all telephone. (Patient 11; telephone)*

Whether remote means of the consultation were actually fit for purpose was a concern. Patients were sometimes asked to upload data or photographs and this posed a challenge:

*But to get a point over, it required me to be very, very precise and to also, in some cases, send photographic evidence because obviously they couldn't see me face-to-face. So there was another skill there as well, which is taking the photograph, uploading it to your system so he could then see what was required rather than seeing me face-to-face. (Patient 11; Telephone)*

Some patient participants felt that remote consultation was not ‘proper care’, that its use was clinician-centred, without consideration for the patient:

*It's not patient centred and it's not patient care …. I think that's one of my biggest issues about the type of consultation people have is the imbalance of power, is that at the moment it's not patient choice. (Patient 10; telephone and video)*

Lack of privacy at home or work and concerns about whether the clinician was somewhere private were raised. Some patients wondered whether the introduction of remote consultation would be permanent and was a cost-saving exercise. If they had to impart results taken at home, they worried about the accuracy and safety of this information. There was a subtle feeling that the balance of power had changed with them having to take more responsibility for their health care. The concerns expressed by patient participants in this study could be seen to be valid fears; they felt that something tangible was lost by changing to remote consultation and that this might be how it will be in the future.

## Discussion

The patient and clinician experiences reported here are a result of a sudden change in service delivery caused by the SARS-CoV-2 pandemic. This is likely to have heightened responses that reflect concerns and fears in both patients and clinicians participating in the study. As in any evaluation, it is possible that those who consented to take part had something to say and our findings should be seen in the light of these factors. Concerns and fears would have been heightened by the pressures of lock-down, furlough, severe illness, pressures on the NHS and fears for personal safety and that of one’s family. However, exploring the responses of the participants in this context is valuable in finding lessons for the future and the legacy that SARS-CoV-2 leaves. There is no indication that any particular patient or clinician group had certain specific trends in their experiences.

The literature discussing patient and clinician experiences reflects the findings of this study. Gilbert et al.^28^ found that clinicians’ experiences of remote consultation uncovered concerns about legal liability, safety, safeguarding and security. Patients share these concerns, feeling greater anxiety and worrying about whether they can effectively convey their symptoms to the clinician.^29^ Our evaluation reflects these concerns specifically in terms of clinician fears about patients ‘getting lost in the system’, security, privacy and safeguarding whilst patients voiced concerns about missed diagnoses and finding the language to communicate their symptoms.

Technical challenges and worries about difficult conversations in this evaluation reflect the work of others^30,31^ and Verma and Kerrison's^32^ rapid review, which highlights concerns about using the technology required for remote consultation. Both patient and clinician participants in our study were concerned about the technical skills needed for remote consultation and were concerned about internet connections, loading up data or measurements (for example blood sugar or blood pressure) and how secure this was. Predictably, many felt unprepared and unsupported.

Aside from practical challenges, the literature supports our participants’ feelings that it is the nature of the consultation that makes it appropriate for remote appointments or otherwise.^33,34^ For diagnosis, physical examination, difficult conversations and bad news and consultations with the vulnerable, the findings of our evaluation reflect the other empirical work to date that remote consultation is not fit for purpose in these contexts^30,35–38^ but could be an adjunct to usual care.^38^

Both patients and clinicians in our evaluation commented how they had to quickly develop new communication skills without any preparation, which may have impacted the effectiveness of the consultation and this has been reported by others.^28,38^ Both patient and clinician participants reported that remote consultation was more successful if they had a pre-existing, trusting rapport with each other; although this is not universal in previous research, the experiences of the participants interviewed for our evaluation told us that remote consultation worked best with those with whom they had an established relationship. This is supported by the systematic scoping review by Thiyagarajan et al.,^20^ who found that studies indicated that not all conditions and contexts are suitable for remote consultation.

The challenges of adapting to new styles of communication and the complexity of this are reported elsewhere^39^ and include the findings by Verma and Kerrison^32^ that patients, in particular, felt the loss of non-verbal communication to be a disadvantage. In our evaluation, clinicians and patients indicated that there was a distinct altered dynamic in remote consultations in that they could not rely on non-verbal means of communication and that, at times, anxiety regarding using remote means affected the content of the consultation and at others made it easier to share something personal. Patient-participants, at times feared the loss of the privacy of the face-to-face consultation and clinicians also fear that this is a risk.^40^

Patient participants told us that they understood why their appointments had been changed to remote means but felt that they had no control over this and that the whole process was very clinician-orientated. They felt that the choice over how their appointments were conducted did not involve them and that some consultations needed to be face to face such as when imparting bad news or for making important decisions. This caused some anxiety and resentment at times and remote consultation was thought to lack patient-centredness. Taking into consideration the context at the time, it is likely that the fear caused by the pandemic, in general, would have affected such concerns.^40^

Clinician participants raised concerns about their personal and professional well-being whilst working remotely. Previous research has found that staff using remote consultation were concerned about their own professional development, citing a lack of guidance on remote consultation and concerns about the therapeutic nature of this means of interaction with patients echoed.^36^ Isolation, uncertainty and a lack of usual leadership and management have been documented by other authors.^41^ Clinicians were often balancing home and work life, many clinicians had children at home, for whom they had to provide home education whilst working and without the benefit of furlough. The sudden move to remote means of seeing their patients meant that they had little time to set up appropriate working arrangements at home and lacked privacy. Many worried about their ability to provide safe care and this is probably more of a comment on the influence of the SARS-CoV-2 pandemic than the actual experience of remote consultation. Given the opportunity to up-skill, to acquire the correct equipment and to prepare adequately, this response may have been very different. Post-pandemic, current indications are that clinicians now see the potential of remote consultation, that their fears have not been played out and that, with more control of the way the process works, they can see the advantages.^42^

For patient-participants concerns that may be considered to be about well-being were voiced as not getting the reassurance from remote consultations that they did face to face; they felt unsupported at times and feared that their consultation might contain inaccuracies due to their inability to communicate effectively with their clinician. It is likely that those who are of older age and also those who are digitally excluded will have more serious concerns.^43,44^ Reassurance that remote consultations are well-planned, resourced, documented, governed and preceded by training would help to give patients and clinicians more confidence for a clear future implementation.^45,46^ Experiences reported in evaluations in other clinical services during the pandemic are valuable and promising.^47–49^

## Conclusions

Clinician and patient experiences reported here were much influenced by the SARS-CoV-2 pandemic, which moved all but acute and emergency consultations online. Whilst patients welcomed the opportunity to avoid travel and parking, clinicians were left feeling that they had suffered emotionally and professionally; they struggled to provide a safe and high-quality experience for patients and had to adapt very quickly to new ways of working. Both clinicians and patients were forced to adapt their communication skills in order to make the consultation effective.

The lessons from the SARS-CoV-2 pandemic for health care have had a global impact. Clinicians have been presented with a huge challenge to continue to provide services by remote means. The patient experiences reported from this evaluation show that the repercussions of this have raised serious concerns about whether remote consultation is currently fit for purpose. The pressures of implementing remote consultation are complex and, twinned with resource restrictions and austerity are a huge challenge for clinicians with the UK NHS strategy to provide a ‘digital first’ option for all consultations is a major challenge for all.

For patients, there is an opportunity to have quick and stress-free consultations as long as this is of a routine or follow-up nature. For more important treatment decisions and for some diagnostic consultations, patients in this study are clear that remote means are unlikely to be appropriate. Where remote means are appropriate, there is a need to educate and train clinicians to get the most from remote consultation and to use it judiciously alongside face-to-face consultation. Patients also need help to understand what is expected from them. Organisationally, decisions need to be made in a contextual way so that consultations are conducted in the right way for the purpose for which they are intended. Organisational support seems to be important to provide clinicians with the means to overcome technical challenges and to find a means of safeguarding both patients and their data. Patients need time and support to prepare for remote consultation and attention needs to be paid to developing the kinds of patient/clinician relationships that make these more patient-centred. There is an opportunity now to make the most of what went well and take time to consider how best to take this forward. There is potential to enhance patient choice and self-management if planned carefully.

Limitations of this study include the single setting of one NHS Trust in the UK and the design of service evaluation which is intended to define or judge current care rather than generate generalisable knowledge. The data were collected by NMAHP fellows who, whilst mentored by experienced researchers and trained, had little experience of interviewing for this purpose. Additionally, it was not clear how many remote consultations patients and clinicians had undergone and thus, levels of experience are unclear.
